# Emotional Contexts Modulate Anticipatory Late Positive Component and Reward Feedback Negativity in Adolescents With Major Depressive Disorder

**DOI:** 10.3389/fpsyt.2020.00358

**Published:** 2020-04-29

**Authors:** Wenhai Zhang, Caizhi Liao, Fanggui Tang, Shirui Liu, Jing Chen, Lulu Zheng, Ping Zhang, Qiang Ding, Hong Li

**Affiliations:** ^1^Mental Health Center, Yancheng Institute of Technology, Yancheng, China; ^2^College of Education Science, Chengdu University, Chengdu, China; ^3^College of Education Science, Hengyang Normal University, Hengyang, China; ^4^Institute for Brain and Psychological Sciences, Sichuan Normal University, Chengdu, China; ^5^Research Center of Brain and Cognitive Neuroscience, Liaoning Normal University, Dalian, China; ^6^Department of Psychological Medicine, Children’s Hospital of Fudan University, Shanghai, China

**Keywords:** adolescents, feedback negativity, late positive component, major depressive disorder, reward anticipation

## Abstract

**Background:**

Neuroimaging research has determined deficits in the dopaminergic circuit of major depressive disorder (MDD) during adolescence. This study investigated how emotional contexts modulate the temporal dynamics of reward anticipation and feedback in adolescents.

**Methods:**

EEG data from 35 MDD and 37 healthy adolescents were recorded when they conducted a gambling task after being presented with emotional pictures.

**Results:**

The results demonstrated that both MDD and healthy adolescents exhibited the largest late positive component (LPC) in positive contexts at the frontal sites and the largest LPC in negative contexts at the central sites; however, MDD adolescents exhibited anticipatory LPC hypoactivation than healthy adolescents. However, MDD adolescents exhibited smaller gain feedback negativity (FN) than healthy adolescents independent of emotional contexts, positively correlating with the trait anhedonia according to the consummatory aspect of the Temporal Experience of Pleasure Scale. In contrast, MDD adolescents exhibited greater FN loss in positive and neutral contexts than healthy adolescents while no difference in FN loss was found between the two groups in negative contexts. Moreover, the FN loss amplitudes negatively correlated with hedonic tone according to the Snaith-Hamilton Pleasure Scale over the past week.

**Conclusions:**

These findings suggest that MDD adolescents exhibited dissociable deficits in reward anticipation and gain or loss feedback that are distinctly modulated by emotional contexts, and they deepen our understanding of the modulation of emotional contexts on the temporal dynamic reorganization of the reward circuit in MDD adolescents.

## Introduction

Adolescence is a critical period of developmental transition in cognitive, affective, and social domains (1). During this period, adolescents undergo dramatic neuroplastic changes in neural structure and function (including the dopaminergic corticomesolimbic circuit) (2), coinciding with a high incidence of mental illnesses (e.g., depression with the subsequent reoccurrence of depressive episodes) (3). Particularly, adolescents in major depressive disorder (MDD) exhibit emotional disturbances and blunted reward sensitivity (4). As MDD impacts not only responses to emotional events, but extends to other cognitive processes (e.g., reward processing) carried out in the context of emotional engagement (5, 6), exploring the interaction between emotional contexts and reward processes is important for the understanding of the reward function reorganization in MDD across adolescence.

It is well-known that contextual factors (e.g., affective state/mood) might promote or subvert goal-directed behaviors (7). According to the appraisal tendency framework (8), contextual emotions generate specific anticipatory effects consistent with underlying appraisal tendencies and carry over to a new situation to alter sequent task performances (9–11). Changes in anticipatory effect might lead to goal reprioritization by modifying the salience of potential gains or loss: positive mood increases the value of reward motivation and activates corticostriatal pathways, including the nucleus accumbens, putamen, and ventromedial prefrontal cortex (9, 12–14). However, these effects are not found during reward outcome, loss anticipation, and loss outcome (14). Following positive mood induction, nucleus accumbens’ activation decreases during reward anticipation (but not receipt) while its connectivity with the dorsolateral prefrontal cortex increases and these effects are modulated by anhedonia (15).

In contrast, negative mood leads to a greater degree of anchor biasing, which reduces reward-processing capacity and makes people avoid loss (16–18). Recently, Park et al. (19) proposed the valence compatibility hypothesis and found that positive emotional valence increases activity in typical reward-processing regions (e.g., ventral striatum) (reflecting additive value effect) while negative valence imposes increased task demands, eliciting conflict processes, which leads to the engagement of regions related to cognitive control (e.g., medial and lateral prefrontal cortex). Thus, how emotional contexts interact with reward processes during the distinct stages of reward anticipation and outcome remains an open question.

Event-related potentials with high temporal resolution easily capture rapid neural responses during different stages of reward processes. Late positive component (LPC) is a slow, positive component that lasts several hundred milliseconds after stimulus onset (20). Generally, positive and negative visual stimuli elicit enhanced LPC amplitudes compared with neutral stimuli (21), reflecting selective attention to motivationally salient stimuli (22). Particularly, during the anticipatory stage, the low positive affect trait was associated with reduced LPC by rewarding images and a blunted threat of eliciting LPC in adult depression, while the negative affect trait was not associated with the magnitude of neural responses to either threatening or rewarding pictures (23). Moreover, Howsley & Levita (24) found that only preadolescents exhibited heightened LPC in the reward block, whereas all preadolescents, adolescents, and late adolescents exhibited LPC potentiation to discriminative stimuli versus control stimuli in the avoidance block.

Feedback negativity (FN) is a negative-going waveform, and it reaches a maximum at the frontocentral sites approximately 300 ms after feedback presentation (25). Reflecting deeper striatal signals or prefrontal responses to changes in striatal activity (26–28), FN is sensitive to outcome evaluation and is correlated with the magnitude of the reward prediction error (29, 30). Following sadness induction, individuals reporting a greater state of sadness exhibited less differentiation between non-rewards and rewards in behavioral and FN responses (31). In contrast, positive emotional contexts enhanced reward-related FN sensitivity to outcomes compared with neutral and negative emotional contexts (9). Moreover, the FN amplitude to gain feedback (but not loss) in MDD adults was related to anhedonia severity (32). In adolescents, depressive symptoms were associated with reduced FN (33, 34), but the opposite trend has also been observed (35). After sadness induction, the reduced FN was also observed in adolescents with high familial risk for MDD (26). Taken together, positive and negative contexts might modulate the temporal dynamics of reward anticipation and outcome evaluation differently in MDD adolescents.

The present study aimed to investigate how emotional contexts interact with reward processes in MDD adolescents. In each trial, a positive, neutral, or negative picture was presented before MDD and healthy adolescents accomplished a simple gamble task, and then they received gain or loss feedback. Based on the appraisal tendency framework and the previous results (19, 23), we expected that positive contexts would increase anticipation-related LPC amplitudes and gain-related FN compared with neutral and negative contexts while negative contexts would amplify loss-related FN compared with positive and neutral contexts. Moreover, if MDD adolescents have impaired capacity to reward processing and greater perceptual biases to avoidance-related cues (24, 32–34), MDD adolescents would exhibit smaller LPC amplitudes and smaller FN amplitudes in gain trials, while MDD adolescents would exhibit larger FN amplitudes in loss trials relative to healthy adolescents.

## Methods

### Participants

In total, we recruited 74 right-handed adolescent students (14–18 years old) from several secondary vocational schools in Dalian City, China. All participants had the normal or corrected vision and provided written informed consent under the approval of their parents. All adolescents completed clinical mini-interviews that were performed separately with adolescents and their parents. MDD adolescents were included if they had a primary MDD diagnosis based on DSM-IV criteria but no other mental disorders and had received no medication treatment for the past 3 months. Healthy adolescents were included if they had no current or past psychiatric diagnoses and serious head trauma. Two healthy adolescents were excluded due to poor EEG quality. Finally, the sample had 35 MDD adolescents (11 males; 15.26 ± 1.41years old) and 37 healthy adolescents (12 males; 15.53 ± 1.36 years old). This study was under the Declaration of Helsinki and was approved by the local ethics committee.

### Stimuli

Two hundred and sixty-four pictures were selected from the Chinese Affective Picture System (36), including 88 positive, 88 neutral, and 88 negative pictures. There were no differences in arousal among positive, neutral, and negative pictures, but there were significant differences in valence among the three picture types (*p* < 0.001), as reported by another sample of 40 healthy adolescents from the same schools. E-prime 2.0 (Psychology Software Tools Inc., Pittsburgh) was used for stimulus presentation and response recording.

### Measures

Beck Depression Inventory-II (BDI-II), a 21-item self-reporting inventory, measures symptoms of depression (past two weeks) (37). The version used for the present study has been validated in the Chinese population (32). Cronbach’s alpha in the current sample was 0.86.

The Temporal Experience of Pleasure Scale (TEPS) was used to assess distinct components of the long-term pleasure experience (including anticipatory and consummatory aspects) (38). This study applied a 20-item Chinese version that was revised from the original English version (38, 39). In the current sample, Cronbach’s alphas for the TEPS-ANT (anticipatory pleasure) and the TEPS-CON (consummatory pleasure) were 0.82 and 0.84, respectively.

The Snaith–Hamilton Pleasure Scale (SHAPS) was used to measure the hedonic tone over the past week (40). This scale contains 14 items and covers four aspects of hedonic experiences, i.e., interests/past times, sensory experience, social interaction, and food/drink. The Chinese version in the current study has good validity in Chinese samples (41). Cronbach’s alpha in our sample was 0.90.

### Procedure

**Figure 1:**
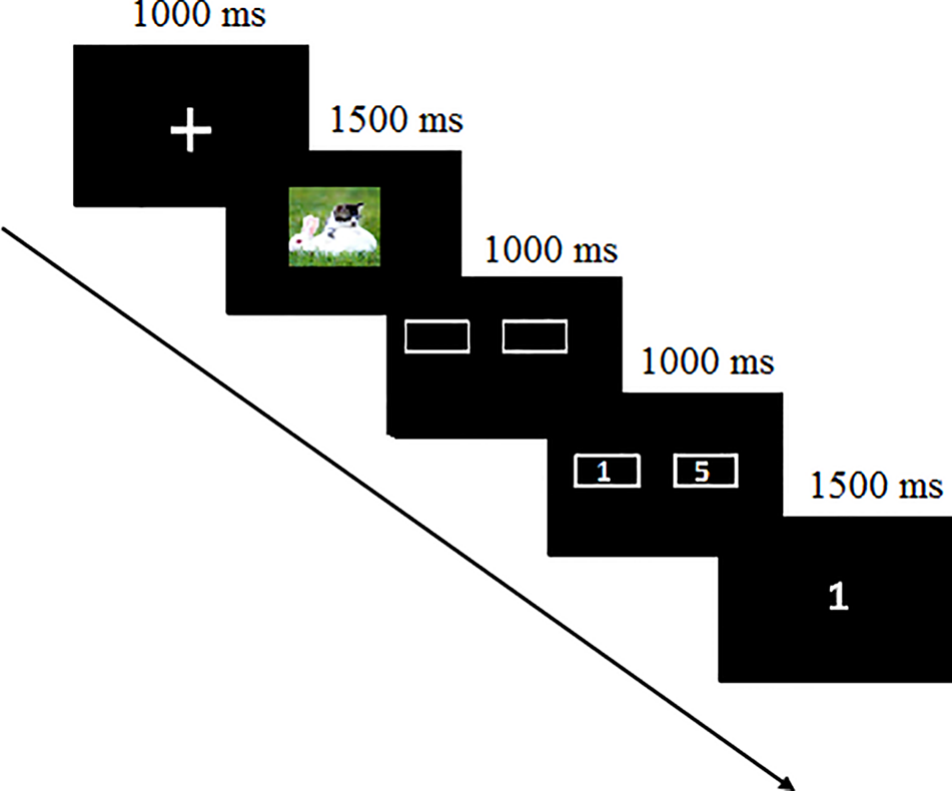
Schematic of the experimental procedure.

In the laboratory, participants were seated in a sound-attenuated, electrically shielded room where they were attached to the sensors. As **Figure 1** shows, after receiving an understanding of the instructions, they conducted a simple gambling task. The task included 240 trials with 80 trials separately for positive, neutral, and negative pictures. At the beginning of each trial, participants were presented with a fixation for 1,000 ms and then a picture for 1,500 ms. Next, two doors appeared on the screen for 1,000 ms when participants were instructed to guess how to choose the door. They were told that they would win 1 or 5¥ in a winning trial or lose 1 or 5¥ in a loss trial. When the doors with the number of 1 or 5 appeared randomly on the left or right side for 1,000 ms, participants pressed the button to choose the door they guessed. The outcome was presented the next screen on which 1 or 5 appeared for a win or −1 or −5 appeared for a loss. During the task, 40 win and 40 loss trials for each picture type were presented in random order. Participants rested 15 s for every 40 trials.

### Data Collection and Statistical Analysis

Continuous scalp EEG was collected from the Active Two system (BioSemi, Netherlands) with a 64-electrode elastic cap arranged according to the 10/20 guidelines. Electrooculogram (EOG) was recorded using four electrodes placed above and below the left eye and at the outer edge of the right eye to monitor horizontal and vertical eye movements. The impedance was brought below 10 kΩ. The data were sampled at 500 Hz with a bandpass of 0.10–100 Hz and a 24-bit A/D converter referenced to the vertex electrode (Cz). EEG data were offline re-referenced to the mean of the two mastoids using Brain Vision Analyzer 2.0 (Brain Product, Munich, Germany). The signals were bandpass-filtered using a second-order digital Butterworth filter (24 dB) of 0.10–30 Hz and adjusted for DC offset. EEG data were visually inspected for artifacts or extreme offsets. Oculomotor artifacts were eliminated using built-in ICA blink templates. Epochs were generated from −200 ms to 1,000 ms following the presentation of the stimuli or feedback and were baseline-corrected using the 200 ms pre-stimulus/feedback interval. Trials with voltage amplitudes over ±50 µV were discarded by an automatic procedure.

The ERP analysis focused on the two stages of anticipation and feedback. During the anticipatory stage, we selected the mean LLP amplitudes of three composite electrodes separately from the frontal (Fz, F1, and F2), frontocentral (FCz, FC1, and FC2), and central (Cz, C1, and C2) electrodes within the 600–800 ms time window. Similarly, during the outcome feedback stage, we selected the mean FN amplitudes of the three composite electrodes separately from the prefrontal (FPz, FP1, and FP2), frontal (Fz, F1, and F2), and frontocentral (FCz, FC1, and FC2) electrodes between 270 and 370 ms.

Repeated-measures analysis of variance (ANOVA) with two groups (MDD and control) × three valences (positive, neutral, and negative pictures) × three regions were conducted separately for LPC, FN in the gain trials, and FN in the loss trials. Independent-sample tests were executed for depressive symptoms and anhedonia severity. The Greenhouse–Geisser correction was used when the variance sphericity assumption was violated. All *post hoc* tests were corrected by the Bonferroni method. Pearson’s correlation analysis was performed to investigate the association between behavioral and ERP data.

## Results

### Behavioral Data

As **Table 1** shows, the independent-sample t-test indicated that MDD adolescents reported significantly higher symptoms of depression as measured by BDI-II than the healthy controls (*t* (71) = 17.41, *p* < 0.001). Moreover, MDD adolescents exhibited lower anticipatory pleasure experience as measured by TEPS-ANT (*t* (71) = 2.96, *p* < 0.01) and higher SHARP scores than the healthy controls (*t* (71) = 5.21, *p* < 0.001). However, there were no sex or age differences between the two groups (*t* (71) = 0.94, 0.61, *p >*0.05).

**Table 1:** Demographic and sample characteristics.

Characteristic	MDD	HCL	Statistic	*p*-value
Age (years)	15.26 ± 1.41	15.53 ± 1.36	*t* = 0.58	0.61
Sex (M/F)	24/11	25/12	χ^2 =^ 0.00221	0.94
BDI-II	33.32 ± 5.71	6.26 ± 1.72	*t* = 17.41	< 0.001
SHARP	28.45 ± 7.21	22.89 ± 5.78	*t* = 5.21	< 0.001
Age of first episode onset (years)	12.62 ± 1.70			
Duration (months)	25.82 ± 6.49			

MDD, major depressive disorder; HCL, healthy controls. BDI, Beck Depression Inventory-II; SHARP, the Snaith-Hamilton Pleasure Scale.

### ERP Data

#### LPC at the Anticipatory Stage

**Figure 2:**
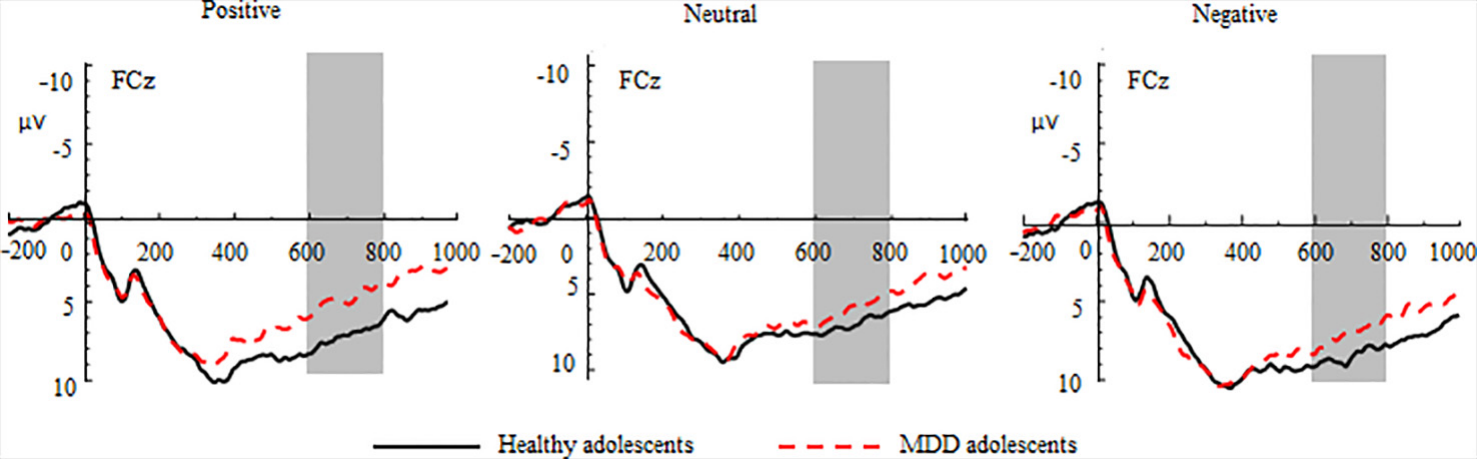
Group waveforms of major depressive disorder (MDD) and healthy adolescents for positive, neutral, and negative pictures during the anticipatory stage at FCz. Late positive components are defined within 600–800 ms.

The three-way repeated-measures ANOVA yielded significant main effects of group (*F*(1, 70) = 4.52, *p* < 0.05, *η^2^* = 0.16), valence (*F*(2, 140) = 29.41, *p* < 0.001, *η^2^* = 0.30), and region (*F*(2, 140) = 8.85, *p* < 0.001, *η^2^* = 0.11) (See **Figure 2** and **Table 2**). The *post hoc* tests indicated that the LPC amplitudes in MDD adolescents were lower than those in healthy adolescents (*p_adj_* = 0.0068); the LPC amplitudes for positive pictures were greater than those for negative pictures, which were larger than those neutral pictures (*p_adj_* = 0.0008); the LPC amplitudes at the frontocentral sites were the greatest (*p_adj_* = 0.0004). Furthermore, there was a significant interaction between valence and region (*F*(4, 280) = 2.97, *p* < 0.05, *η^2^* = 0.10). The *post hoc* tests indicated that at the frontal sites, the LPC amplitudes for positive pictures were greater than those for negative and neutral pictures (*p_adj_* = 0.0274); at the central sites, the LPC amplitudes for negative pictures were larger than those for positive and neutral pictures (*p_adj_* = 0.0165); at the frontocentral sites, there were no differences in LPC amplitudes for the three type pictures (*p_adj_ >*0.05). No other significant effects were observed.

**Table 2:** Results of the 3-factorial repeated-measures analysis of variance (ANOVA).

Dependent variables	Variables	*df*	*F* value	*p*-value
Anticipatory LPC	Group	1, 70	4.52	0.0132*
	Valence	2, 140	29.41	0.0005***
	Region	2, 140	8.85	0.0042**
	Group × valence	2, 140	2.51	0.2964
	Group × region	2, 140	2.21	0.3872
	Valence × region	4, 280	2.97	0.0312*
	Group × valence × region	4, 280	2.08	0.1245
FN in gain trials	Group	1, 70	8.51	0.0076**
	Valence	2, 140	1.68	0.5843
	Region	2, 140	23.46	0.0006***
	Group × valence	2, 140	2.31	0.3745
	Group × region	2, 140	10.32	0.0052**
	Valence × region	4, 280	2.10	0.1205
	Group × valence × region	4, 280	1.68	0.5026
FN in loss trials	Group	1, 70	14.87	0.0026**
	Valence	2, 140	3.97	0.0231*
	Region	2, 140	8.91	0.0039**
	Group × valence	2, 140	8.47	0.0061**
	Group × region	2, 140	1.97	0.5167
	Valence × region	4, 280	2.12	0.1201
	Group × valence × region	4, 280	1.49	0.6781

LPC, late positive component; FN, feedback negativity. *p < 0.05, **p < 0.01, ***p < 0.001.

#### FN at the Feedback Stage

**Figure 3:**
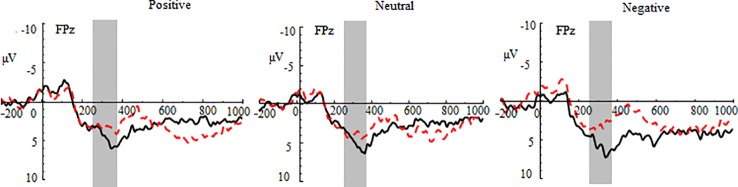
Group waveforms of major depressive disorder (MDD) and healthy adolescents for positive, neutral, and negative pictures during the gain feedback stage at FPz. Feedback negativity is defined between 270 and 370 ms after the presentation of feedback.

**Figure 4:**
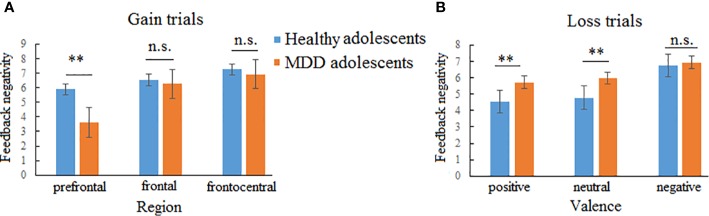
Two-way interactions of feedback negativity (FN) in gain and loss trials. **(A)** Interaction between group and region in gain trials indicating that major depressive disorder (MDD) adolescents exhibited lower FN amplitudes than healthy adolescents at the prefrontal region while no group difference was found at the frontal and frontocentral regions. **(B)** Interaction between group and valences indicating that MDD adolescents exhibited larger FN amplitudes for positive and neutral pictures than healthy adolescents while no group difference was found for negative pictures. n.s., not significant. **p < 0.01.

In the gain trials, the three-way repeated-measures ANOVA yielded significant main effects of group (*F* (1,70) = 8.51, *p* < 0.01, *η^2^* = 0.31) and region (*F* (2,140) = 23.46, *p* < 0.001, *η^2^* = 0.25; See **Figure 3** and **Table 2**). The *post hoc* tests indicated that MDD adolescents exhibited lower FN amplitudes than healthy adolescents (*p_adj_* = 0.0036); the FN amplitudes were greatest at frontocentral sites and smallest at the prefrontal sites (*p_adj_* = 0.0048). Additionally, there was a significant interaction between group and region (*F* (2,140) = 10.32, *p* < 0.01, *η^2^* = 0.21) (See **Figure 4**). The *post hoc* tests indicated that MDD adolescents exhibited lower FN amplitudes at the prefrontal sites (*p_adj_* = 0.0036) while there were no differences in FN amplitudes at the frontal and frontocentral sites (*p_adj_ >*0.05).

**Figure 5:**
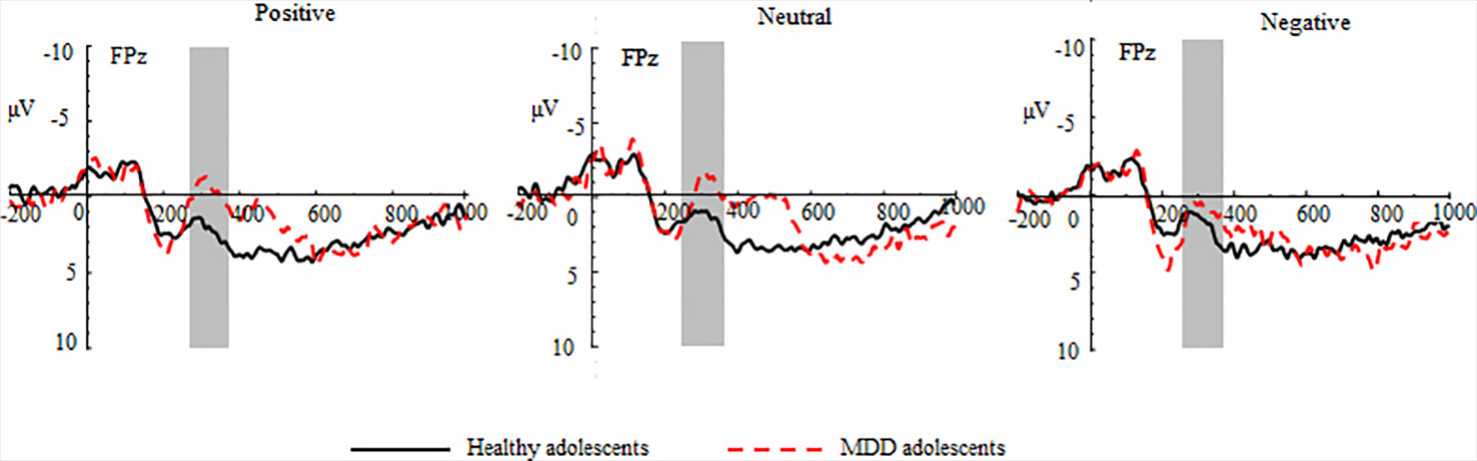
Group waveforms of major depressive disorder (MDD) and healthy adolescents for positive, neutral, and negative pictures during the loss feedback stage at FPz.

In the loss trials, the three-way repeated-measures ANOVA revealed significant main effects of group (*F* (1,70) = 14.87, *p* < 0.01, *η^2^* = 0.44), valence (*F* (2,140) =3.97, *p* < 0.05, *η^2^* = 0.11), and region (*F* (2,140) = 8.91, *p* < 0.01, *η^2^* = 0.11) (See **Figure 5** and **Table 2**). The *post hoc* tests indicated that MDD adolescents exhibited greater FN amplitudes than healthy adolescents (*p_adj_* = 0.0028); the FN amplitudes for negative pictures were more pronounced than those for positive and neutral pictures (*p_adj_* = 0.0128); the FN amplitudes at the frontocentral sites were the highest (*p_adj_* = 0.0051). Moreover, there was a significant interaction between group and valence (*F* (2,140) =8.47, *p* < 0.01, *η^2^* = 0.21) (See **Figure 4**). The *post hoc* tests indicated that MDD adolescents exhibited more pronounced FN amplitudes for positive and neutral pictures than healthy adolescents (*p_adj_* = 0.0027, 0.0045), while we detected no differences in FN amplitudes for negative pictures between the two groups (*p_adj_* = 0.1827).

### Correlation Analysis

The correlation analyses indicated that the BDI scores were negatively correlated with LPC amplitudes for positive (*r* = −0.26, *p* < 0.05) and negative pictures (*r* = −0.25, *p* < 0.05) but not for neutral pictures (*r* = −0.06, *p >*0.05). In the gain trials, the TEPS-CON scores were positively correlated with the FN amplitudes for positive, negative, and neutral pictures (*r* = 0.31, 0.31, 0.34, *p* < 0.01). In the loss trials, the SHARP scores were positively correlated with the FN amplitudes for positive, negative, and neutral pictures (*r* = 0.24, 0.24, 0.25, *p* < 0.05).

## Discussion

This study used LPC and FN as neural indices to examine how emotional contexts modulate reward anticipation and outcome evaluation in MDD adolescents. The behavioral results indicated that MDD adolescents had a lesser anticipatory pleasure experience (TEPS-ANT) and higher SHARP scores than healthy adolescents. The ERP results indicated that at the anticipatory stage MDD adolescents exhibited smaller LPC amplitudes than healthy adolescents; at the feedback stage, MDD adolescents exhibited smaller FN amplitudes for the gain trials than healthy adolescents while MDD adolescents exhibited greater FN amplitudes for the loss trials than healthy adolescents. Consistent with the previous fMRI and ERP results (32–34), these observations suggest that MDD adolescents exhibited impaired capacity toward reward processing (i.e., anhedonia) and greater perceptual biases toward negative outcomes. Moreover, the emotional contexts modulated LPC and FN in adolescents, discussed as follows, which is important implication for our understanding of the temporal dynamical organization and development of brain function during adolescence.

Many researchers have found that positive mood facilitates reward anticipation motivation, including increased corticostriatal circuit activation (9, 12–14) and anticipatory LPC amplitudes (24). In line with these results, our study found that the anticipation-related LPC amplitudes in positive picture contexts were larger than those in negative and neutral picture contexts at the frontal sites. In contrast, the negative picture contexts elicited larger LPC amplitudes than the positive and neutral picture contexts at the central sites. On the one hand, consistent with the appraisal tendency framework (8), positive mood further enhanced the value of reward motivation while negative mood increased avoidant motivation, which reduced the value of reward anticipation (16–18). On the other hand, Park et al. (19) found that reward trials by positive emotion did not enhance activity differently anywhere in the adult brain. Inconsistent with this, our results suggest that as LPCs seem to be modulated by anticipatory top-down signals from the frontoparietal neural network (42, 43), the adolescent brain is undergoing functional reorganization and exhibits cortical differences in top-down processing approach and avoidant motivation. For example, the previous adult study indicated that positive and negative stimuli activated different functional networks (44). Future research in adolescents should further verify the possibility of interaction between valences and cortical regions.

During reward receipt, the group × region interaction on FN amplitudes reached significance but independent of emotional contexts, indicating that MDD adolescents exhibited smaller FN amplitudes than healthy adolescents only at the prefrontal sites. Consistent with our results, the previous ERP and behavioral studies also found that emotional stimuli are processed independent of reward (45, 46). This might be attributed to the more pronounced gain-related FN amplitudes reflecting a phasic increase in dopaminergic activity in the reward pathways, which partly underpins the associations between gain-related FN and trait measures; for example, extraversion has higher scores on a dopaminergic function and is associated with enhanced FN waves compared with introversion (47). We also found that gain-related FN amplitudes were positively correlated with TEPS-CON scores (reflecting anhedonia trait). Another possibility is that emotional contexts are task-irrelevant and have a long internal with feedback presentation, which leads to the disappearance of the carryover effect. However, this did not explain why loss-related FN amplitudes are modulated by emotional contexts as discussed in the following.

Notably, we observed a significant group × valence interaction on loss-related FN amplitudes, indicating that MDD adolescents exhibited greater FN amplitudes in positive and neutral contexts than healthy adolescents while no FN difference was observed between the two groups in negative contexts. These results seemed to be consistent with the valence compatibility hypothesis (19). During loss feedback that leads to an aversive signal, positive contexts elicited conflict processes with loss feedback consumption, which makes frontal control engage with conflict regulation. The previous results indicated that MDD individuals have impaired corticostriatal connectivity (e.g., dorsolateral prefrontal cortex and striatum) following positive mood induction (12, 15). Thus, MDD adolescents seemed to be incapable of modulating potential conflicts, which further amplifies the aversive effects of losing money. In contrast, there was no valence mismatch between negative contexts and transient loss manipulation, so that MDD adolescents exhibited the same FN amplitudes as healthy adolescents. Moreover, the loss-related FN amplitudes were positively correlated with the SHARP scores, possibly reflecting anhedonia. Overall, these results suggest that emotional contexts did not appear to modulate gain-related FN, reflecting a lower dopamine function (trait anhedonia) in MDD adolescents, whereas emotional contexts might modulate loss-related FN, partly reflecting anhedonia from disrupted regulating function in corticostriatal circuits.

The limitations must be mentioned as follows. First, it should be acknowledged that this study had a small sample. Future studies should perform a power analysis to estimate the effect size and select a larger sample to verify the results. Second, although there were 40 trials for gain and 40 loss trials for loss, there were insufficient trials to separate ±1 or 5 Y trials. Therefore, future studies should add the number of gains and losses to separately analyze whether rewards of different sizes have an impact on FN amplitudes.

In summary, MDD adolescents exhibited dissociable deficits in reward anticipation and outcome processing that are modulated by emotional contexts. Anticipation-related LPC hypoactivation in MDD adolescents might reflect aberrant functional reorganization in top-down cortical networks that show different sensitivity to positive and negative contexts. Emotional contexts did not modulate reduced gain-related FN in MDD adolescents, which might reflect trait-like reward deficits in MDD adolescents. However, positive (but not negative) contexts seem to amplify loss-related FN, revealing impaired conflict-regulating function from the lateral and medial prefrontal cortex in MDD adolescents. These observations provide important evidence for our understanding of the temporal dynamic reorganization of the reward functions in MDD adolescents during distinct emotional contexts.

## Data Availability Statement

The data that support the findings of this study are available from the corresponding author upon reasonable request.

## Ethics Statement

This study was in accordance with the Declaration of Helsinki and was approved by the Ethics Committee of Liaoning Normal University. Written informed consent to participate in this study was provided by the participants’ legal guardian/next of kin.

## Author Contributions

LZ, QD, SL, PZ, and WZ designed and performed the study and analyzed the data. WZ wrote the manuscript. CL, FT, HL, and JC reviewed and modified the manuscript.

## Funding

This study was supported by the National Natural Science Foundation of China (31470997 and 81171289) and Jiangsu Provincial Social Science Foundation of China (19JYD009).

## Conflict of Interest

The authors declare that the research was conducted in the absence of any commercial or financial relationships that could be construed as a potential conflict of interest.
